# A low morbidity surgical approach to the sheep femoral trochlea

**DOI:** 10.1186/1471-2474-14-5

**Published:** 2013-01-03

**Authors:** Patrick Orth, Henning Madry

**Affiliations:** 1 Center of Experimental Orthopaedics and Osteoarthritis Research, Saarland University, Homburg/Saar, Germany; 2 Department of Orthopaedic Surgery, Saarland University Medical Center, Homburg/Saar, Germany

**Keywords:** Cartilage, Sheep, Trochlea, Patella, Luxation, *In vivo*

## Abstract

**Background:**

The ovine stifle joint is an important location for investigations on the repair of articular cartilage defects in preclinical large animals. The classical medial parapatellar approach to the femoral trochlea is hazardous because of the high risk of postoperative patellar luxation. Here, we describe a low morbidity surgical exposure of the ovine trochlea without the necessity for intraoperative patellar luxation.

**Methods:**

Bilateral surgical exposure of the femoral trochlea of the sheep stifle joint was performed using the classical medial parapatellar approach with intraoperative lateral patellar luxation and transection of the medial patellar retinaculum in 28 ovine stifle joints. A low morbidity approach was performed bilaterally in 116 joints through a mini-arthrotomy without the need to transect the medial patellar retinaculum or the oblique medial vastus muscle nor surgical patellar luxation. Postoperatively, all 72 animals were monitored to exclude patellar luxations and deep wound infections.

**Results:**

The novel approach could be performed easily in all joints and safely exposed the distal two-thirds of the medial and lateral trochlear facet. No postoperative patellar luxations were observed compared to a postoperative patellar luxation rate of 25% experienced with the classical medial parapatellar approach and a re-luxation rate of 80% following revision surgery. No signs of lameness, wound infections, or empyema were observed for both approaches.

**Conclusions:**

The mini-arthrotomy presented here yields good exposure of the distal ovine femoral trochlea with a lower postoperative morbidity than the classical medial parapatellar approach. It is therefore suitable to create articular cartilage defects on the femoral trochlea without the risk of postoperative patellar luxation.

## Background

Preclinical models of articular cartilage repair are of paramount importance to translate experimental approaches into the clinical situation [1]. The sheep or goat stifle joint is one preferred model for those studies [2-4] as it combines unique advantages over other species, such as similarities in the repair capacity of articular cartilage defects and similar biomechanical properties including long bone dimensions and body weight to humans [5,6]. The mechanical loading environment occurring in sheep and goats is well understood [7-9] and they are easier to handle than pigs or horses, including anesthesia [10].

From a human articular cartilage repair standpoint, the trochlea is an important location to create cartilage defects [11], since the clinical outcome of defects at this anatomical site is unfavorable compared with lesions in the femoral condyles [12]. Moreover, the sheep trochlea is plane with a large surface, making it an ideal site to study articular cartilage repair in a standardized manner.

The surgical exposure of the trochlea by the classical medial parapatellar approach involves the intraoperative luxation of the patella. While easy to perform in patients [13], a significant rate of postoperative patellar luxations may occur in sheep or goats when applying the classical approach. Such patellar luxations are based on the different anatomy of the ovine and caprine stifle joint when compared to the human knee: in extension, the patella is located proximal to the trochlea and glides within the trochlear groove only in flexion of the stifle joint. This special feature of the more posteriorly angled trochlea, in combination with a relatively small lateral femoral condyle, makes complications very likely if a medial parapatellar incision and lateral patellar luxation is chosen. In addition, the ovine medial retinaculum is mostly stronger and more difficult to repair after transection. Besides, even if no patellar luxation occurs, osteoarthritis may frequently result from using the classical approach, possibly jeopardizing surgical results considerably [14].

Here, we describe a low morbidity surgical exposure of the ovine femoral trochlea with reduced risk for patellar luxation.

## Methods

### Study design

Surgical exposure of the femoral trochlea of the sheep stifle joint was performed by the same surgeon (HM) using either a standard medial parapatellar approach with luxation of the patella (n = 14 sheep) or a novel mini-arthrotomy without intraoperative patellar luxation (n = 58). All sheep (n = 72) were allowed full weight-bearing and full range of motion immediately postoperatively. Animals operated for previous [15,16] or unpublished studies of experimental articular cartilage repair served as subjects to describe the surgical approaches.

### Animals

Healthy, skeletally mature, Merino ewes aged between 2 and 4 years (mean body weight [BW], 70 ± 20 kg) received water *ad libitum* and were fed a standard diet. Osteoarthritis was excluded on preoperative X-rays of the stifle joints. All animal experiments were conducted in accordance with the national legislation on protection of animals and the National Institutes of Health (NIH) Guidelines for the Care and Use of Laboratory Animals (NIH Publication 85–23, Rev 1985) and were approved by the local governmental animal care committee.

### Anesthesia

Following a 12-hour fast, animals were sedated with 2% Rompun (Bayer, Leverkusen, Germany) at 0.05 mg/kg BW and endotracheally intubated after intravenous administration of 20 ml of 2% propofol (AstraZeneca, Wedel, Germany) and carprofen (1.4 mg/kg BW; Pfizer, Berlin, Germany). Anesthesia was maintained by inhalation of 1.5% isoflurane (Baxter, Unterschleißheim, Germany) and intravenous administration of propofol (6–12 mg/kg BW/h). At the day of the operation and the first or second postoperative day, respectively, animals routinely received analgesia (carprofen; 1.4 mg/kg BW; Pfizer, Berlin, Germany) and antibiotics (amoxicillin clavulanate; 30 mg/kg BW; Pfizer).

### Classical medial parapatellar approach

Prior to development of the novel less-invasive surgical approach, exposure of the ovine femoral trochlea has been performed by the use of the classical medial parapatellar approach in 28 stifle joints in 14 sheep.

According to the report of Allen *et al.*[17], a medial parapatellar skin incision was made from 5 cm proximal to the patella to a point 5 cm distal to the tibial tubercle. With the proximal incision, the oblique medial vastus muscle often had to be incised to allow for luxation of the patella. The joint capsule was opened medial and parallel to the patellar tendon with transection of the medial patellar retinaculum. The patella was luxated laterally and retracted by the use of a Hohmann retractor. For closure of the capsule, absorbable sutures were applied (Vicryl; size 2; Ethicon, Norderstedt, Germany). The subcutaneous tissue and the skin incision were closed using Vicryl size 2/0 and 2, respectively. Simple interrupted suture patterns were applied for all anatomical layers. Aluminium bandage spray was applied to the wounds.

### Novel less-invasive approach

The novel approach to the ovine femoral trochlea was applied in 116 arthrotomies in 58 sheep.

#### Animal positioning and draping

**Figure 1:**
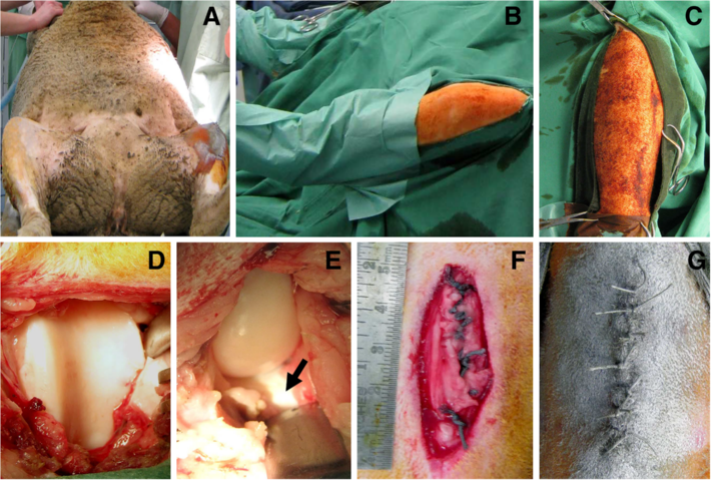
**Key procedures of the novel less-invasive surgical approach.** The sheep were placed in a supine position (**A**). with linen sheets onto the abdomen to extend the sterile area (**B**). Full extension of hip and stifle joints by axially pulling both hindlimbs during wrapping is crucial to later avoid a possible shifting of the drapes during intraoperative joint mobilization (**B**). Fenestrated linen sheets are used to cover the proximal, lateral and medial borders, leaving open a triangular operative site (**C**). Following the slightly oblique skin incision (length 4–5 cm) and arthrotomy, exposure of the distal two thirds of the lateral and medial facet of the femoral trochlea is achieved (**D**). This preserves the oblique medial vastus muscle and the medial patellar retinaculum and retains the patella in a proximal position without the need for its intraoperative surgical luxation (Figure 2). Each femoral condyle (**E**) as well as the anterior third of each meniscus (**E**; arrow) can also be safely exposed when applying different degrees of stifle joint flexion. For closure of the capsule, non-absorbable sutures were used (**F**). The surgical wounds (**F**) were closed in layers by simple interrupted suture patterns. Finally, aluminium bandage spray was applied (**G**).

The sheep were placed in a supine position with both hindlimbs untied (Figure 1A). The skin over both stifle joints was shaved consecutively using a gross and a fine electric shaver. No tourniquet was applied, limbs were aseptically (Braunol, Braun, Melsungen, Germany) prepared for surgery. Linen sheets were placed onto the abdomen of the sheep to extend the sterile area (Figure 1B). The hindlimbs were then put through fenestrated linen sheets, allowing covering of the proximal, lateral and medial borders and leaving open a triangular operative site (Figure 1C). The fenestrated sheets were secured with towel clips. Impermeable plastic drapes (70 × 70 cm) were used to wrap lower limb and claw. Full extension of hip and stifle joints during wrapping of the lower leg is crucial to avoid later shifting of the drapes during intraoperative joint mobilization (Figure 1B). Adhesive tapes were used to fixate the plastic drape around the claws. Stockinettes are a more expensive alternative for the wrapping of the lower limb.

#### Surgical approach

**Figure 2:**
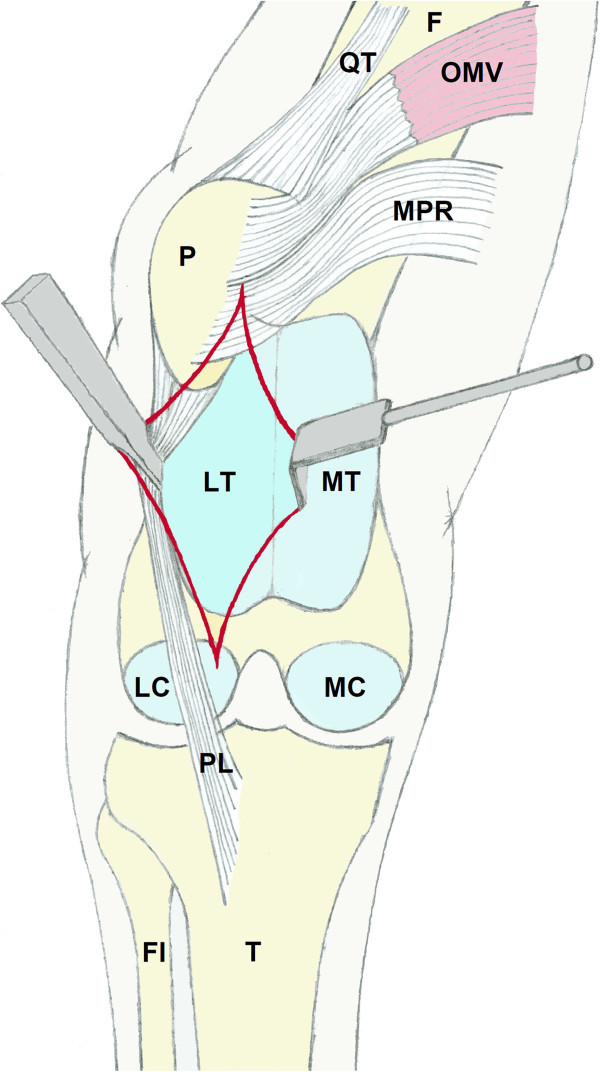
**Schematic drawing of the surgical anatomy of the novel low morbidity approach.** The described less-invasive surgical approach allows for a minimally invasive exposure of the distal two thirds of the medial and lateral trochlear facet in sheep. It preserves the oblique medial vastus muscle as well as the medial patellar retinaculum that would have to be transected using the classical medial parapatellar approach. Especially as no intraoperative patellar luxation is needed, the risk for postoperative patellar luxations is decreased compared to the classical approach. Moreover, flexion of the stifle joint allows for a good exposure of the medial and lateral condyle as well as the anterior third of both menisci. The skin incision is depicted in dark red. F: femur; QT: quadriceps tendon; OMV: oblique medial vastus muscle; MPR: medial patellar retinaculum; P: patella; LT: lateral trochlear facet; MT: medial trochlear facet; LC; lateral femoral condyle; MC: medial femoral condyle; PL: patellar ligament; FI: fibula; T: tibia. For ease of visualization, the remaining parts of the quadriceps muscle as well as other capsular structures and parts of the ovine anatomy are not shown.

Important landmarks include the patella, the patellar ligament, and the tibial tubercle (Figure 2) [17]. Under constant pull on the hindlimb to ensure full extension of the hip and stifle joint, a straight skin incision of 4–5 cm length was made, extending from a point 1 cm medial of the inferior pole of the patella towards the tibial tubercle. This oblique incision takes in account that the trochlear groove does not run in parallel to the femoral shaft but is orientated at an angle of 20 ± 5 from proximolateral to distomedial [17]. Utmost care was taken to prevent the oblique medial vastus muscle from any damage in the proximal part of the wound. The subcutaneous tissue was divided in the line of the skin incision by the use of an electrocautery, ensuring hemostasis. The medial border of the patellar tendon was exposed and the joint was entered by cutting through the joint capsule. Importantly, this distal location of the arthrotomy preserves the medial patellar retinaculum (Figure 2). Since the synovium and the joint capsule are intimately related [17], the capsular incision also opened the synovium. Small Weidtlaner wound retractors served to facilitate exposure. Care was taken not to damage the cartilaginous joint surface of the trochlea during arthrotomy. For better exposure of the femoral trochlea, the parapatellar fat pad was either retracted with a Langenbeck retractor or partially resected. A small Hohmann retractor was then placed between the patellar ligament close to the inferior patellar pole and the proximolateral femoral condyle (Figure 2). This retractor was used to lift the patella and retain it in a proximal position without intraoperative surgical luxation. The additional axial pull on the hindlimb and full extension of stifle and hip joints now allowed exposure of the distal two thirds of the lateral and medial facet of the femoral trochlea (Figure 1D). Most importantly, by the use of this approach, the patella was neither everted nor luxated out of the patellofemoral joint. Instead, it is tilted laterally and displaced proximally by the use of a Hohmann retractor and maximal extension of the hip and stifle joint. In addition, the medial patellar retinaculum is not transected, minimizing the risk for postoperative patellar luxations. Furthermore, the medial and lateral femoral condyle (Figure 1E, Figure 2) as well as the anterior third of both menisci (Figure 1E) can also be exposed easily using this approach when applying different degrees of stifle joint flexion.

#### Wound closure

The surgical wounds (length 4–5 cm; Figure 1F) were closed in layers. First, the medial extensor mechanism (capsule and patellar tendon) was meticulously reconstructed. This suture is of paramount importance with regard to the prevention of postoperative patellar luxations. Therefore, we chose the non-absorbable Ethibond suture (size 6; Ethicon, Norderstedt, Germany) to ascertain a biomechanically stable reconstruction of the extensor mechanism. Additional Ethibond sutures (size 3) served for tight closure of the capsule (Figure 1F). The intermediate soft tissue layer, the subcutaneous tissue, and the skin were readapted using absorbable sutures (Vicryl; size 2, size 2/0, size 2, respectively; Ethicon, Norderstedt, Germany). Simple interrupted suture patterns were applied for all anatomical layers. Finally, an aluminium bandage spray was applied to the wounds (Figure 1G).

### Postoperative monitoring

All 58 animals were continuously monitored over 6 months to exclude postoperative patellar luxations, deep wound infection, or empyema.

#### Patellar luxation

The animals were examined daily over the first 5 weeks and weekly for the remaining observation period by adspection for clinical signs of patellar luxation such as abnormalities in hindleg carriage, stance, or lameness. Additionally, with the sheep in a sitting position, thorough palpation of the joints was conducted and they were put through a range of motion. Digital pressure was applied to the medial border of the patella to test for its stability and exclude luxation. The different grades of patellar luxation in sheep are given in Table 1. X-ray was performed only in the case of uncertainty upon clinical examination.

**Table 1:** Grades of patellar luxation in sheep

**Grade**	**Patella at examination**	**Luxation**	**Reposition**	**Reluxation**	**Lameness**	**Bone deformities**
I	reduced	manual by digital pressure	spontaneous	rare	mild	seldom
II	reduced in extension and luxated in flexion	manual by digital pressure or spontaneous in flexion	manual by digital pressure or spontaneous in extension	upon manipulation	resolvable skipping lameness	sometimes
III	luxated	spontaneous	manual by digital pressure	frequent	severe	often
IV	luxated	permanent	not possible	permanent	crouching stance	very often

Patellar luxations in sheep are evaluated according to the grading scales developed for small animals such as cats and dogs [50,62]. Diagnosis can be made upon clinical examination; radiographic analysis is not necessary. Clinical signs at adspection include abnormalities in gait or hindleg carriage with the stifle joint flexed, lameness, locking of the affected limb in extension, and -especially in bilateral luxations- crouching, bowlegged or knock-kneed stance.

#### Deep wound infections and empyema

All stifle joints were examined clinically for redness, hyperthermia, swelling, and secretion and body temperature was measured daily over the first 3 weeks and weekly for the remaining observation period.

## Results

### Classical medial parapatellar approach

**Figure 3:**
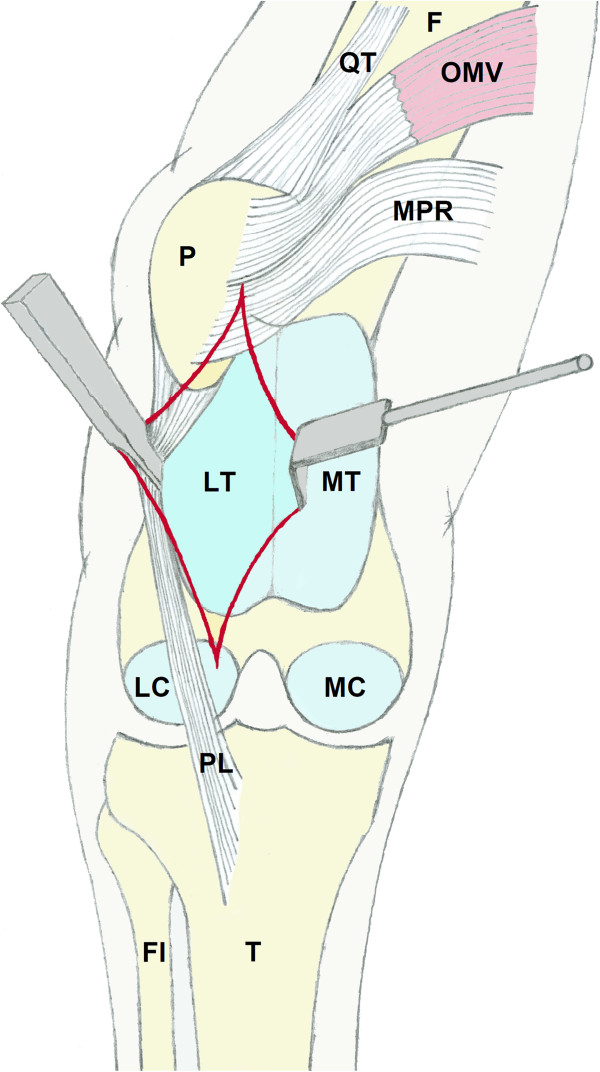
**Anteroposterior radiographic view of a right ovine stifle joint with a grade III patellar luxation.** The luxated patella (arrowheads) can be identified proximolateral to the lateral femoral epicondylus (star), dislocated outside of the patellar groove.

Prior to development of the less-invasive surgical approach, exposure of the ovine femoral trochlea had been performed bilaterally using the standard medial parapatellar approach with intraoperative lateral luxation of the patella in 28 stifle joints (n =14 animals). Inadvertent postoperative luxation of the patella was frequently observed in 7 stifle joints (25%) (n = 5 animals); two animals (14%) suffered from bilateral patellar luxations. Anteroposterior X-rays were performed in two animals to verify the clinical diagnosis (Figure 3). The postoperative interval until luxation was of 14–21 days. Luxations were grade II (n = 2) and grade III (n = 5), indicating that manual reposition was always feasible (Table 1). Therefore, animals were first treated with anti-inflammatory medication (carprofen) for one week. As lameness worsened in all animals, surgical stabilization of the patella was performed by revision with capsular and retinacular imbrication. Of note, the previously applied absorbable sutures were not detectable any more within the subcutaneous tissue or at the level of the capsule at the time of revision surgery (3–4 weeks postoperatively), indicating their significantly faster absorption compared to humans. One animal with bilateral luxations did not survive the revision surgery due to complications during anesthesia. Out of the five revised stifle joints, four exhibited re-luxation of the patella with necessity for sacrifice of 2 sheep. Other severe complications such as deep wound infections or empyema were not observed.

### Novel less-invasive approach

The surgical procedure was performed bilaterally in 116 joints (n = 58 sheep). Exposure of the trochlea was always successful. Anesthesia was uneventful. There were no intraoperative complications. Surgical time was between 15 and 20 min per joint. The sheep recovered quickly, as indicated by their rise already at 1–2 hours and the full weightbearing on both operated hindlimbs 24 hours postoperatively. Upon clinical examination, no signs of lameness (assessable in bilaterally operated animals only upon full weightbearing), deep wound infections, or empyema were observed at any time point. Skin incisions healed 2 weeks after surgery. No patellar luxations were observed in any of the sheep operated with the novel surgical approach.

## Discussion

The authors describe a less-invasive surgical approach to the ovine stifle joint, allowing for a low morbidity exposure of the femoral trochlea without the need for intraoperative patellar luxation. This approach was applied in 116 stifle joints and is technically easy, safe, and yields good exposure of the distal two thirds of the trochlea. Surgical benefits include the preservation of important structures, such as the medial patellar retinaculum, the oblique medial vastus muscle, and of patellar blood supply. Importantly, postoperative patellar luxation never occurred. This approach therefore may be valuable to create articular cartilage defects on the ovine femoral trochlea.

The sheep or goat stifle joint is a key animal model reflecting many features of the human knee, including the relative sizes of articulating bones, the straight leg axis, or the existence of cruciate ligaments, menisci, asymmetrical collateral ligaments, and a bicondylar distal femur [2-4,6]. Compared to other large animals such as horses, sheep and goats have the advantages of easy handling and cost effectiveness [10]. However, goats can prove more difficult to house than sheep [18]. Besides, although the articular cartilage is thicker in goats (up to 1.9 mm) than in sheep (0.3-0.7 mm) [19], goats are more susceptible to spontaneous osteoarthritis [18]. Therefore, we here applied sheep rather than the goat model.

Regarding articular cartilage defects, the sheep has been used as animal model for partial- [20] and full-thickness [21-23] chondral and osteochondral defects [11,24,25]. Several studies also used the ovine stifle joint to test implants [26] and as a model for osteoarthritis [27,28], cruciate ligament reconstruction [29,30], or meniscus repair [31]. However, the classical medial parapatellar arthrotomy includes surgical patellar luxation, unnecessarily increasing the risk for postoperative complications such as patellar luxation. With a view of animal welfare and protection, a less-invasive surgical approach with reduced risk for patellar luxation is required.

To date, most studies on articular cartilage repair in sheep used the medial femoral condyle [21-25,32-41], perhaps because of its uncomplicated surgical exposure. Despite an improved histological grading of chondral defects at the ovine trochlea compared with the condyle after 8, 10, and 12 weeks [42], the trochlea is underrepresented in these investigations [11,34]. This may in part be due to the decreased thickness of normal cartilage at the trochlea compared with the femoral condyles [19] or the tibial plateau [43], although proteoglycan levels of trochlear and condylar cartilage are similar [44,45]. Yet, due to its plane and large cartilaginous surface, the ovine trochlea is suitable for cartilage repair studies. The here described surgical approach not only allows for a safe exposure of this important anatomical site, but also gives access to the medial and lateral femoral condyle and both menisci.

The preclinical sheep model is prone to rather high postoperative complication rates which might not always be reported. These include wound dehiscence or infections due to non-anatomical wound closures ignoring the layered structure of the soft tissue, the reduced antiseptic environment caused by the nature of the animal model itself, missing routing in draping, insufficient surgical experience and knowledge of the anatomical structures, or extended operation times [46]. Besides, complication rates in the sheep model are additionally increased by the fact that reduced loading of the operated hindlimb can only be achieved by extended measures such as applying splints, harnesses [47], plaster casts [48], or hanging the animals in slings [49].

In the present report, patellar luxations only occurred following the classical medial approach with intraoperative patellar luxation and transection of the medial patellar retinaculum. Thus, although considered a standard approach for clinical and preclinical purposes [17], we strongly advise against this erroneous surgical technique in future translational animal experiments and in veterinary surgery. This serious complication may additionally be provoked by the specific anatomy of the ovine patellofemoral joint (small lateral femoral condyle, patella located outside and proximal to the trochlear groove in extension, proximolateral to distomedial angle of the trochlear groove [17]), together with the high loading forces resulting from an unprotected rise from the lying down position [49]. Besides, the high luxation rate following the classical approach may possibly be ascribed in part to a genetic predisposition for patellar luxation in sheep [50]. Altogether, these factors may favour a unilateral experimental set-up, allowing for at least one pain-free hindleg to facilitate postoperative rising and standing.

Clinical signs of patellar luxation include persistent, abnormal hindleg carriage with the stifle joint flexed, lameness, and -especially in bilateral luxations- crouching, bowlegged or knock-kneed stance (Table 1), and may usually be perceived upon postoperative weightbearing (circa 24 hours after surgery). The suspected diagnosis can be affirmed by clinical examination: When luxated, the patella is palpable laterally, with a grinding sensation when being mobilized and a snapping sound upon reduction. Although not necessary routinely, additional radiological examination by X-ray (anteroposterior (Figure 3) or flexed dorsoproximal-dorsodistal radiographic views [50]) may confirm the diagnosis. In this report, the initial treatment of these grade II-III luxations was non-operatively, but as lameness and non-weightbearing worsened, soft tissue revision surgery (capsular and retinacular imbrication) was performed. In order to preserve the femoral trochlea, osseous reconstruction procedures initially developed for the canine model [50] such as trochleoplasty [51] or tibial tuberosity transposition [52] were avoided here. Intriguingly, the postoperative rate of re-luxations was of 80%. In good agreement, failure rates of 80% and 50% have been reported for the surgical treatment of patellar luxations in llamas [52] and dogs [53], respectively. Thus, although the mild temperament and relatively small body size of sheep generally renders them amenable for the operative treatment of orthopedic problems [51,52,54], we do not recommend revision surgery in the case of postoperative patellar luxations in this animal model.

Interestingly, absorbable suture materials were degraded much faster in sheep than in humans. For the closure of the joint capsule, the relatively high concentrations of inflammatory cytokines [32] and activated matrix metalloproteinase-2 [55] of the ovine synovial fluid might explain this finding, while the elevated mean body temperature of sheep (38-40°C) compared to humans [56,57] may contribute to a generally faster degradation of absorbable foreign materials. Therefore, although applied in routine protocols for knee arthrotomies in patients and in various sheep studies [36,42,48,58-60], Vicryl and other absorbable sutures are inadvisable for the reconstruction of the biomechanically important extensor mechanism. In veterinary medicine, monofilament and thicker absorbable sutures are used successfully for this purpose [61]. Here, we applied non-absorbable suture materials, but the potential risk of postoperative fistula formation has to be acknowledged and further evaluated in future investigations. Regarding the suture pattern, interrupted sutures are preferable compared to continuous sutures [61].

## Conclusions

Patellar luxation is a frequent complication when the classical medial parapatellar arthrotomy is performed in sheep. Its diagnosis can be made be simple physical examination -X-ray is not necessary for its confirmation. The presented approach to the femoral trochlea avoids the need for intraoperative surgical patellar luxation. It is technically easy, safe, and yields excellent exposure of the distal part of the trochlea. Having used this technique in 116 arthrotomies, patellar luxation never occurred. However, the surgeon still must be mindful of this severe complication, especially when the proximal incision is carried our too far, extending into the oblique medial vastus muscle or transecting the medial patellar retinaculum.

## Competing interests

The authors declare that they have no competing interests.

## Authors’ contributions

PO and HM were equally involved in conception and design of the study, surgical treatment and animal care, acquisition, analysis and interpretation of the data, and drafting of the manuscript and revising it critically for important intellectual content. Both authors read and approved the final manuscript.

## Authors’ information

PO is working as a resident at the Department of Orthopaedic Surgery, Saarland University Medical Center, and as a postdoctoral fellow at the Center of Experimental Orthopaedics, Saarland University. HM is working as a registrar at the Department of Orthopaedic Surgery, Saarland University Medical Center, and is the director of the Center of Experimental Orthopaedics, Saarland University.

## Pre-publication history

The pre-publication history for this paper can be accessed here:

http://www.biomedcentral.com/1471-2474/14/5/prepub
